# Hillslope memory of sequential earthquakes

**DOI:** 10.1126/sciadv.aeh3921

**Published:** 2026-09-02

**Authors:** Xin Wang, John D. Jansen, Li Chen, Lanxin Dai, Kushanav Bhuyan, Chengyong Fang, Lorenzo Nava, Filippo Catani, Xuanmei Fan

**Affiliations:** ^1^ State Key Laboratory of Geohazard Prevention and Geoenvironment Protection, Chengdu University of Technology, Chengdu, China.; ^2^ GFÚ Institute of Geophysics, Czech Academy of Sciences, Prague, Czechia.; ^3^ Department of Geography, King’s College London, London, UK.; ^4^ King’s Institute for Artificial Intelligence, King’s College London, London, UK.; ^5^ Department of Geosciences, University of Padova, Padova, Italy.

## Abstract

Large earthquakes can trigger landslides that reshape mountain landscapes within minutes, yet their geomorphic legacy can unfold over millennia. A fundamental question is whether a major earthquake leaves hillslopes in a less or more stable state for the next. Here we exploit two sequential earthquakes (2013 and 2022) at close to the same location near Lushan, China, to quantify how the first event preconditions hillslope failure during the second. The 2013 earthquake produced a dual effect: Regional susceptibility declined as unstable material was removed, yet localized coseismic damage weakened a different population of hillslopes that later failed in 2022. These opposing outcomes generate three damage-recovery pathways of hillslope response that define how mass is redistributed and mechanical strength reorganizes according to a prolonged “hillslope memory” with implications for the landscape evolution of mountain belts. Hillslope response to sequential earthquakes is fundamentally path dependent, demanding landslide hazard assessments that explicitly incorporate history.

## INTRODUCTION

Mountain landscapes evolve via interactions between slow, continuous processes and abrupt catastrophic disturbances (*1*, *2*). Among these disturbances, large earthquakes stand out as exceptionally powerful agents capable of triggering widespread landsliding and reorganizing entire hillslope systems within minutes (*3*). Yet, their geomorphic impact persists long after the shaking stops (*4*, *5*). Directly visible is the disruption of surface drainage across hillslopes locally stripped of soil (*6*), while in the shallow subsurface (<100 m), earthquakes inflict mechanical damage in the form of micro/macrocracking over a wide range of scales. This damage is succeeded over time by mechanical closure of cracks and slower fluid-mediated healing or sealing that may either strengthen rock via cementation or eventually weaken it via focused weathering (*7*, *8*). This postseismic recovery, or relaxation, imparts a form of “hillslope memory”: an inherited mechanical influence of prior seismic damage that potentially governs how hillslopes respond to subsequent perturbations. The timescale of relaxation has been investigated via seismic interferometry that shows changes in seismic velocity follow an exponential or logarithmic decay function, reverting to background values in a period of days to years (*8*, *9*). Although the role of memory is widely acknowledged in seismology and landscape evolution studies (*2*, *10*), the influence of an inherited mechanical state on landslide susceptibility remains scarcely quantified and poorly understood (*9*). Yet, this inheritance may impose an outstanding degree of control on how hillslopes respond to shaking and perhaps even the rate at which landscapes evolve over geological timescales.

All existing coseismic landslide prediction models treat each earthquake as an experiment isolated in time, implicitly assuming that the landscape is temporally static and that hillslope failure depends exclusively on external forcing, typically seismic intensity (*11*, *12*). However, recent observations challenge this assumption: Analysis of two successive earthquakes in Aotearoa New Zealand shows that later coseismic failures tend to concentrate in zones that previously experienced strong shaking, a response best explained by cumulative, damage-driven weakening of hillslopes (*13*). This field evidence echoes the observed heightened activity of rainfall-triggered landsliding following large earthquakes, possibly due to lowered frictional thresholds (*14*, *15*). Conversely, laboratory experiments demonstrate that modest seismic shaking can densify granular materials and increase frictional resistance, thereby stabilizing hillslopes against subsequent perturbations (*16*). Hence, two competing outcomes arise, weakening versus strengthening, although the case for each is bound up with challenges in reproducing boundary conditions in the laboratory and with finding satisfactory empirical data. This lack of robust and systematic testing leaves unresolved the fundamental question: Does an earthquake render hillslopes less or more stable and how does this inherited state regulate future failures?

### Two major earthquakes: Nine years and 12 km apart

**Fig. 1.**
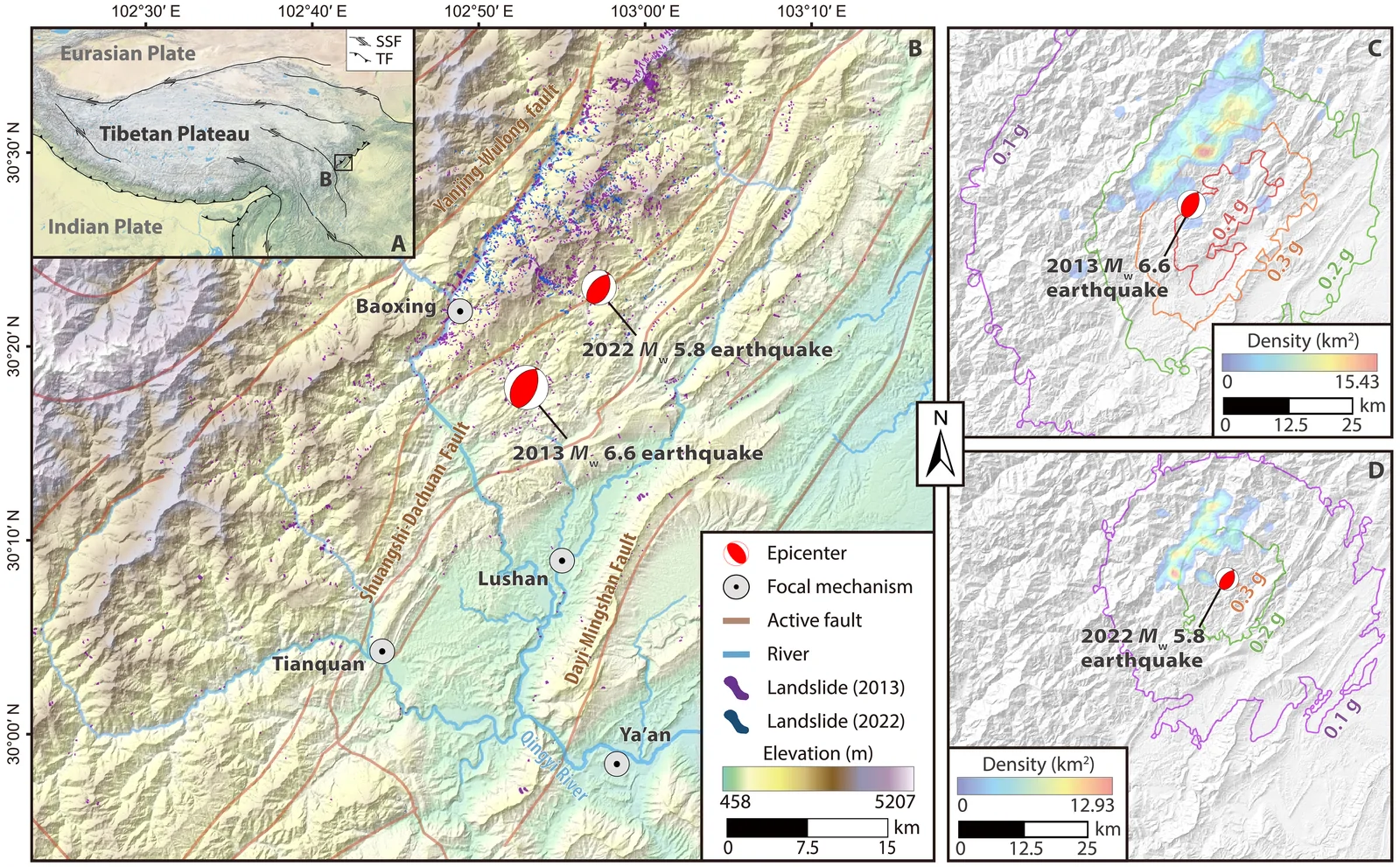
Study area and coseismic landslides of the two Lushan earthquakes. (**A**) Tectonic setting of the southeastern Tibetan Plateau and the thrust-dominated Longmenshan Fault Zone, adapted from “TibetanPlateauTectonics.png” by Mikenorton, Wikimedia Commons (https://commons.wikimedia.org/wiki/File:TibetanPlateauTectonics.png), licensed under CC BY-SA 3.0 (https://creativecommons.org/licenses/by-sa/3.0/); changes include the removal of selected fault and tectonic-unit labels, simplification of the fault legend, and addition of the study-area locator. SSF, strike-slip fault; TF, thrust fault. (**B**) Mapped landslides, epicenters and focal mechanisms associated with the two Lushan earthquakes. The 2013 event originated on a northwest-dipping blind thrust east of the SD fault and caused 217 fatalities and 11,470 injuries; the 2022 event ruptured a southeast-dipping blind thrust and resulted in 4 fatalities and 42 injuries. (**C** and **D**) Coseismic landslide density maps of the 2013 and 2022 events.

The 2013 moment magnitude (*M*_w_) 6.6 and 2022 *M*_w_ 5.8 Lushan earthquakes provide a rare well-controlled natural experiment for isolating the role of hillslope memory in coseismic landsliding. The two events, ∼12 km apart, ruptured adjacent blind thrust structures within the same imbricate system of the southern Longmenshan Fault Zone (*17*, *18*) and thus affected nearly identical topographic, lithological, and climatic settings (Fig. 1, A and B). Despite their close proximity, the earthquakes differed in rupture geometry and shaking distribution, creating distinct sequences of mechanical disturbance imposed on the same hillslopes (*19*). By integrating decade-scale, high-resolution landslide inventories with cross-event susceptibility modeling and ground-surface movement observations (between events), we quantify how hillslopes transitioned from the mechanically altered state left by the 2013 earthquake to the response observed after the 2022 event. This framework allows us to disentangle the relative roles of shaking intensity, inherited damage, and post-seismic recovery.

## RESULTS

### Landslides triggered by the 2013 and 2022 Lushan earthquakes

We developed landslide inventories for both earthquakes by integrating five published polygon-based datasets (*20*–*24*) with additional remote-sensing change detection and manual interpretation. The 2013 earthquake triggered 2820 landslides with a total area of 22.11 km^2^, whereas the 2022 earthquake produced 1090 landslides covering 5.06 km^2^. Both events exhibit a coherent spatial pattern: Landslides cluster between the Shuangshi-Dachuan (SS-DC) and Yanjing-Wulong (YJ-WL) faults, where steep relief and hanging-wall amplification enhance shaking (*25*). In 2013, the highest landslide density (15.43 landslides/km^2^) was observed in the meizoseismal zone along the SS-DC fault, whereas in 2022, the highest density (12.93 landslides/km^2^) occurred along the right bank of the Qingyi River (Fig. 1, C and D). Of particular note is a total of 390 landslides occurred at overlapping locations in both earthquakes, covering 1.06 km^2^, representing 4.78% of the 2013 and 20.90% of the 2022 landslide area. These recurrent failures provide a critical basis for evaluating how inherited damage and residual strength governed hillslope response to repeated seismic loading (table S1).

### Environmental controls of coseismic landslides

To characterize the mechanical conditions governing coseismic landslides, we examined 13 conditioning factors encompassing the following attributes: topographic (altitude, slope, aspect, and curvature), geological (lithology, soil type, and distance to fault), hydrological (distance to river and topographic wetness index), ecological (normalized difference vegetation index and land cover), and seismological [peak ground acceleration (PGA)] (Supplementary Text). These variables collectively capture the structural configuration, material properties, and external forcing that shape hillslope stability during seismic events (*11*).

In both earthquakes, landslides clustered along active faults and river valleys, where rock fracture and fluvial incision reduce shear resistance (*26*, *27*). Failure frequency increased with slope and altitude before declining at high values, consistent with thresholds documented in steep mountain belts (*28*). Lithological and land-cover controls were also evident: Landslides occurred preferentially in metamorphic terrains, lithosol (stony) soils, and shrubland units, reflecting the influence of weak regolith and limited root reinforcement (*29*, *30*). These spatial trends outline a consistent set of preconditioning factors underpinning hillslope failure in the Lushan region. Among all factors, PGA exhibited the clearest distinction between the two events. In 2013, 78.3% of landslides occurred within 0.16 to 0.34 g, whereas 88.6% of the 2022 landslides clustered within the narrower range of 0.16 to 0.24 g. This shift reflects both the weaker shaking intensity of the 2022 earthquake and a landscape whose susceptibility had evolved since 2013. By encapsulating structural, geomorphic, and seismic controls, these factors provide insights into coseismic landslide mechanisms and also serve as robust inputs for susceptibility models (text S1 and fig. S1).

### Landslide susceptibility modeling

**Fig. 2.**
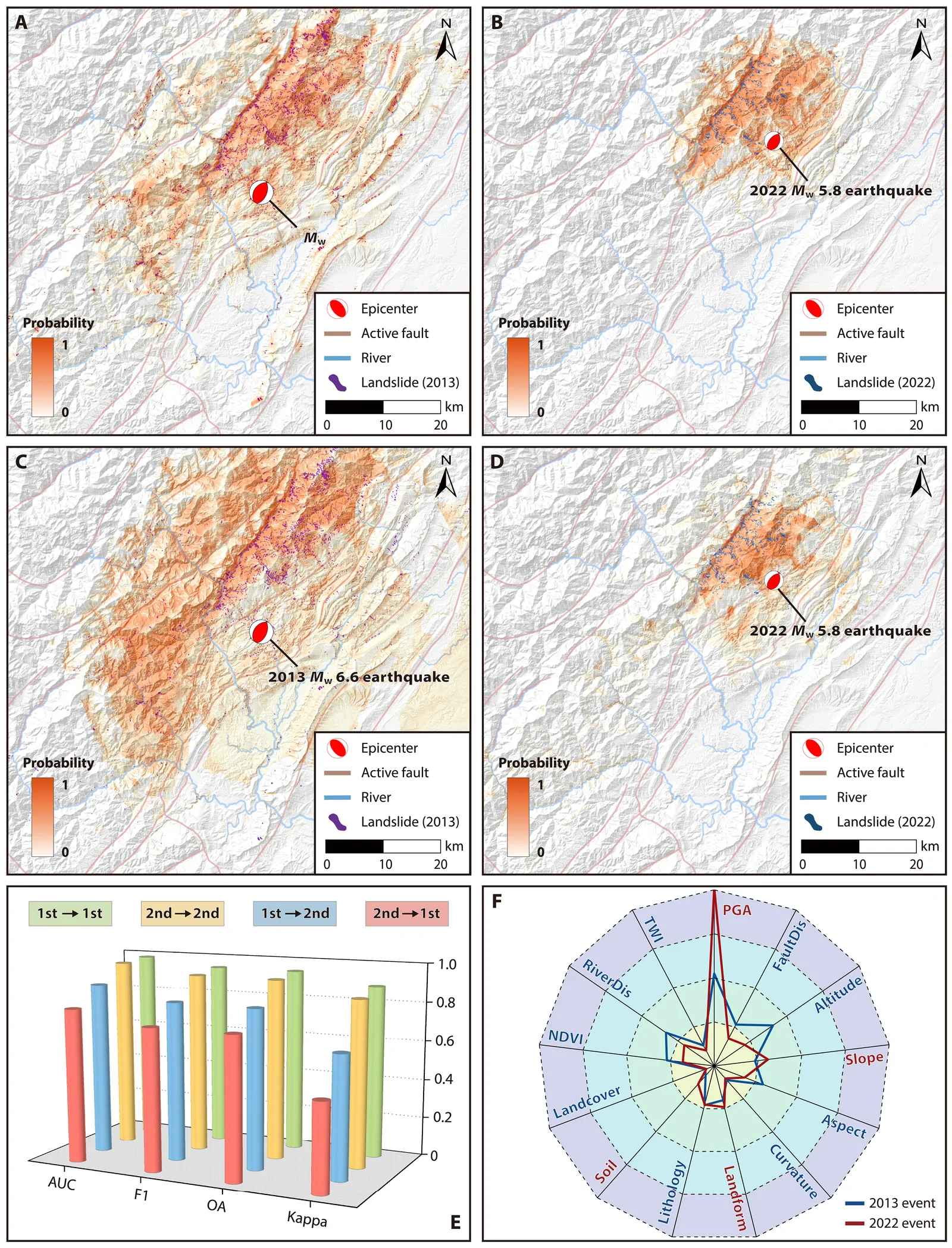
Comparison of coseismic landslide predictions for the 2013 and 2022 earthquakes. (**A** and **B**) Within-event predictions for the 2013 and 2022 earthquakes, in which Random Forest models are trained and tested on landslides from the same event. (**C** and **D**) Cross-event predictions, where the 2022-trained model is applied to 2013 landslides and the 2013-trained model is applied to 2022 landslides. (**E**) Accuracy metrics for all prediction strategies. Arrows in the legend denote model transfer direction (training event → prediction event). (**F**) Normalized importance of conditioning factors in the two event-specific models. Red and blue labels indicate increased or decreased model dependence on a given factor in 2022 relative to 2013, reflecting an evolved mechanical state of the landscape. FaultDis, distance to fault; RiverDis, distance to river.

To access landslide susceptibility for each earthquake, we trained random forest (RF) models using balanced positive and negative samples for the 2013 and 2022 events. Within-event predictions, models trained and tested on landslides from the same earthquake, accurately reproduced mapped failure patterns (Fig. 2, A and B), yielding area under the curve (AUC) values > 0.97 and *F*_1_ score and overall accuracy (OA) > 0.92 (Fig. 2E). These results confirm that, when each event is considered independently, the susceptibility models effectively capture the dominant environmental controls on coseismic landsliding.

To determine whether these controls remained consistent across earthquakes, we conducted cross-event experiments by applying the 2022-trained model to the 2013 coseismic landslides and vice versa (Fig. 2, C and D). Despite substantial spatial overlap between the two inventories, cross-event prediction accuracy declined in both directions (Fig. 2E). The decline was asymmetric: The 2022-trained model performed substantially worse when applied to the 2013 event than the reverse case. This asymmetry indicates that the factor-response relationships learned from each event differ meaningfully, suggesting that the underlying susceptibility structure was not identical between the earthquakes.

To identify the origin of these divergent cross-event predictions, we compared the importance of each conditioning factor between the two susceptibility models (Fig. 2F). Although the overall predictor ranking remained broadly consistent, the normalized importance of PGA exhibited the largest gain (+15.68 ± 1.18%) in the 2022-trained model compared to the 2013 model, indicating a stronger model dependence on seismic forcing in the later event. This shift shaped the contrasting susceptibility patterns. When the 2022-trained model was applied to the 2013 event, its heightened sensitivity to PGA generated expanded high-susceptibility zones wherever strong shaking occurred. Steep terrain in the western mountains was assigned substantially higher susceptibility and even the gentle eastern and southern forelands developed continuous belts of moderate susceptibility. In contrast, applying the 2013-trained model to the 2022 event yielded a marked contraction of medium- and high-susceptibility classes, particularly north of the YJ-WL fault and south of the SS-DC fault. This pattern reflects the reduced weighting of PGA in the 2013-trained model, which dampens the spatial signature of the 2022 shaking field and suppresses susceptibility in areas heavily affected during the later event.

Together, the cross-event performance differences, variable-importance shifts, and contrasting susceptibility spatial patterns demonstrate that the statistical controls on coseismic landsliding were not identical for the two earthquakes. The landscape upon which each model was trained had changed, laying the foundation for subsequent analysis of how these differences arose from the interaction between shaking intensity and inherited hillslope conditions.

### Seismic shaking associated with landslide occurrence

**Fig. 3.**
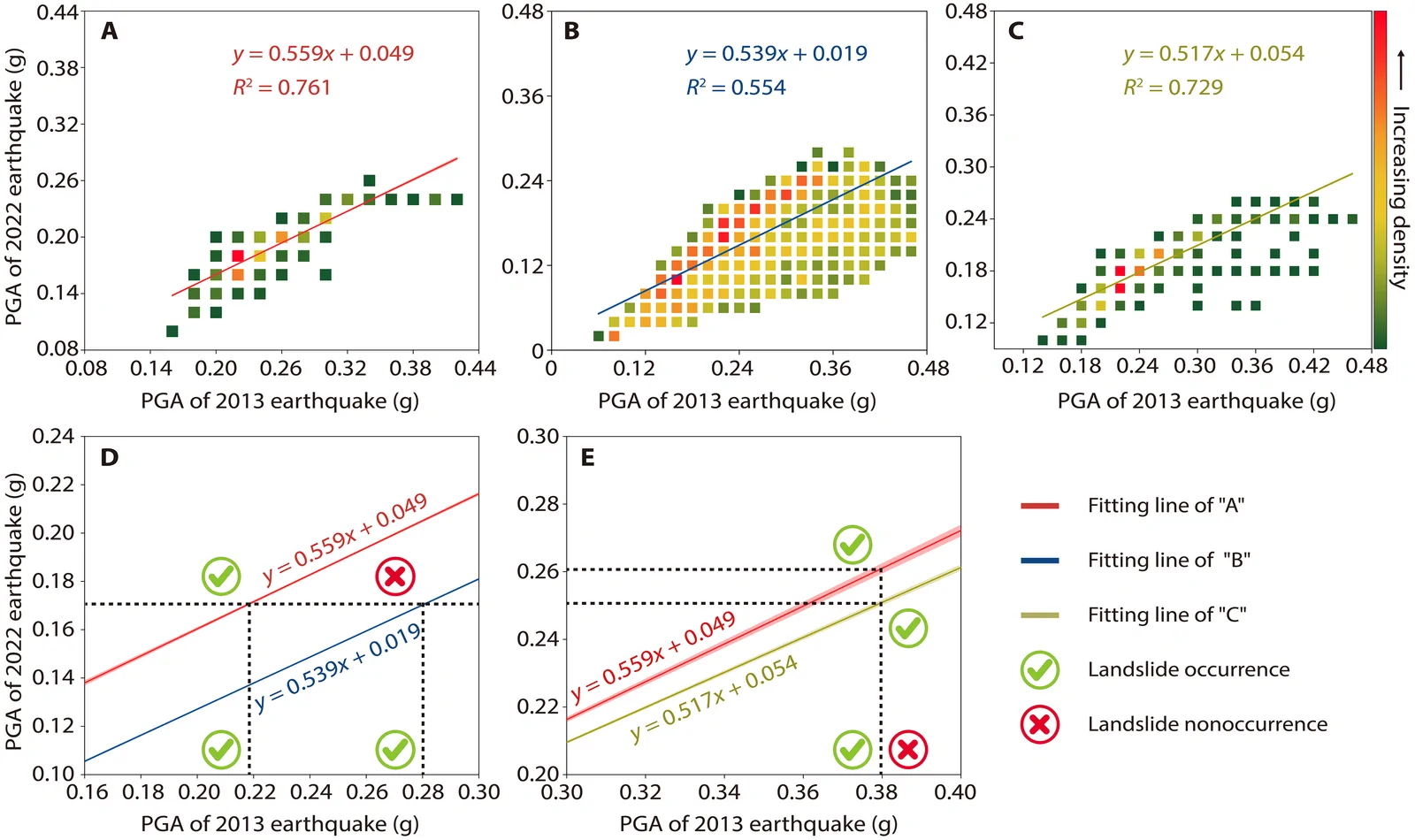
Seismic shaking associated with coseismic landslides during the two earthquakes. PGA distributions for hillslopes with failures in both earthquakes (**A**), only in 2013 (**B**), and only in 2022 (**C**). Scenario 1 compares hillslopes that failed only in 2013 with those with failures in both events (**D**). Scenario 2 compares hillslopes that failed only in 2022 with those with failures in both events (**E**). Shaded intervals represent 95% confidence bounds for linear fits, which are shown as empirical summaries of the association between 2013 and 2022 PGA values for each hillslope category within the observed data range.

Although the 2013 event generated higher overall PGA, differences in shaking intensity alone cannot explain the contrasting spatial patterns of coseismic landslides between the two earthquakes. Given that ground shaking was the only external trigger, we examined how hillslopes with different failure histories responded to repeated shaking. Landslides were grouped into three categories: (i) hillslopes that failed only in 2013, (ii) hillslopes that did not fail in 2013 but failed for the first time in 2022, and (iii) hillslopes that failed in both 2013 and 2022. To isolate the influence of inherited hillslope conditions, we compared PGA relationships across these three categories (Fig. 3, A to C).

We designed two comparative scenarios. Scenario 1 contrasted hillslopes that failed only in 2013 with those that failed in both events (Fig. 3D). At an equivalent PGA in 2022 (e.g., ∼0.17 g), the fitted relation indicates that hillslopes with failures in both events had failed under lower shaking in 2013 (∼0.22 g) than hillslopes that failed only once in 2013 (∼0.28 g). This contrast shows that hillslopes with failures in both earthquakes had remained more susceptible to renewed failure than hillslopes that failed only in 2013: For the same later shaking, they had failed under weaker initial shaking.

Scenario 2 compared hillslopes that failed only in 2022 with those that failed in both events (Fig. 3E). For the same PGA in 2013 (e.g., ∼0.38 g), hillslopes with failures in both events required stronger shaking (∼0.26 g) than newly failed hillslopes (∼0.25 g) in 2022. This contrast shows that under comparable initial shaking, hillslopes that had already failed in 2013 became less susceptible to later failure, requiring stronger shaking for renewed failure in 2022, whereas hillslopes that remained intact in 2013 failed in 2022 under slightly weaker shaking.

These analyses reveal divergent post-seismic trajectories among hillslopes subjected to sequential earthquakes. Some became increasingly failure-prone and collapsed under smaller subsequent loading, while others became less responsive after losing their most unstable components. These contrasting behaviors provide the physical context for understanding the susceptibility differences identified in the modeling analysis.

### Post-seismic hillslope movement revealed by remote sensing

**Fig. 4.**
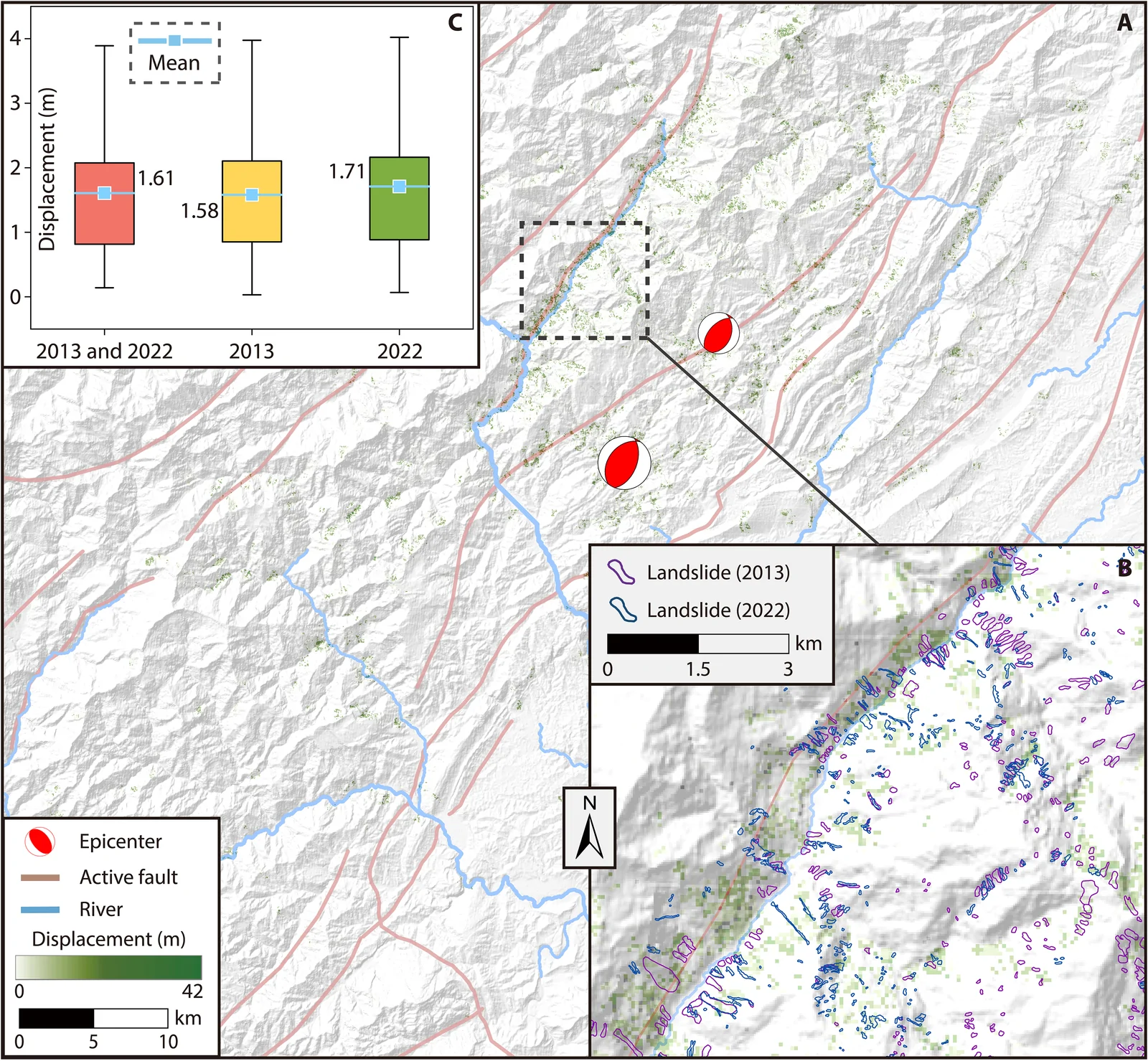
Interseismic ground-surface movement between the 2013 and 2022 earthquakes. (**A**) Cumulative displacements derived from Landsat 8 images using COSI-Corr. (**B**) Enlarged view of hillslope displacements where coseismic landslides were most densely concentrated. (**C**) Interseismic displacements for hillslopes classified by landslide pathway: failures only in 2013, failures only in 2022, and failures in both events.

As we describe above, the landscape continues to adjust mechanically between major earthquakes. Ground-surface movement measured in the years between the two Lushan events (interseismic movement) provides an independent way of assessing how hillslopes evolve mechanically over time. Using Landsat 8 imagery combined with COSI-Corr (*31*), we derived cumulative displacements for all hillslopes across the study area between 2013 and 2022 (Fig. 4A). Pronounced movement was concentrated in the zone between the SS-DC and YJ-WL faults, coincident with the core area of coseismic landsliding, whereas hillslopes that did not experience coseismic failures exhibited markedly less interseismic movement (Fig. 4B). Mean displacements for the three landslide categories further reveal systematic differences in adjustment. Hillslopes that failed only in 2022 show the highest interseismic displacements, followed by hillslopes with failures in both earthquakes, whereas hillslopes that failed only in 2013 display the lowest displacements (1.71, 1.61, and 1.58 m, respectively; Fig. 4C). These gradients are consistent with the interpretation that hillslopes left intact after the first earthquake continued to deform and may have remained more mechanically fragile, while hillslopes that failed in 2013 had already removed part of their most unstable material and consequently exhibited reduced interseismic deformation.

Independent Sentinel-1 observations processed with Persistent Scatterer and Distributed Scatterer InSAR techniques yielded consistent deformation patterns consistent with the optical analyses (fig. S2). These observations support the idea that sustained, internally driven hillslope deformation persists long after the cessation of strong shaking. We can see the landscape undergoes a prolonged phase of mechanical reorganization following major earthquakes, consistent with the time-dependent slope conditions documented in the cross-event susceptibility and PGA relationship analyses.

## DISCUSSION

### From statistical asymmetry to three damage-recovery pathways

**Fig. 5.**
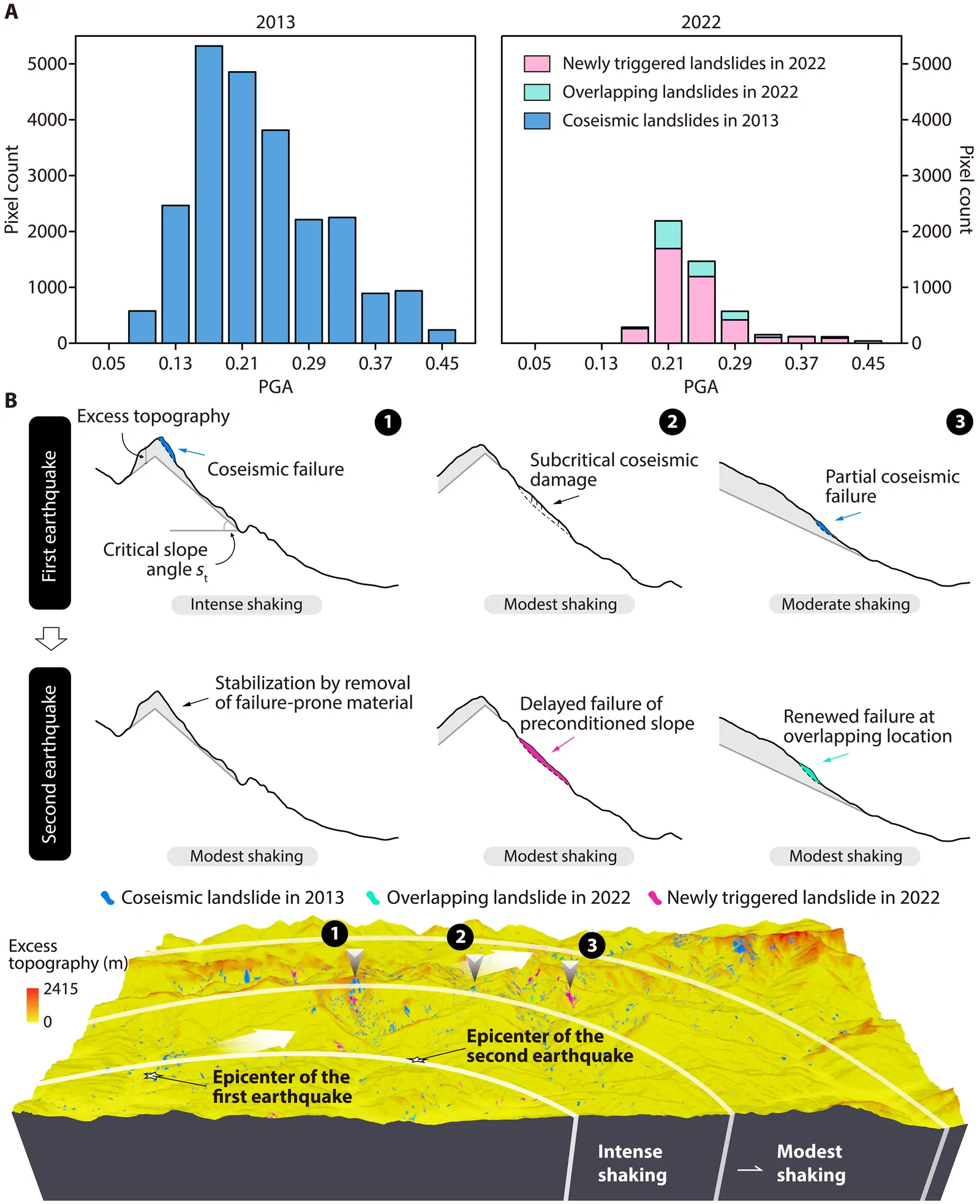
Hillslope responses and landscape adjustment under sequential earthquakes. (**A**) Pixel-wise comparison of coseismic landslide areas that experienced equivalent PGA in the two earthquakes. (**B**) Three damage-recovery pathways of hillslopes subjected to repeated seismic forcing: (1) full exhaustion and stabilization after strong shaking, (2) delayed failure following subcritical damage, and (3) partial failure and mobilization of damaged remnants. Excess topography used in the base map quantifies the rock mass protruding above a specified critical slope angle (i.e., oversteepened hillslopes) and thus highlights zones of relief prone to landsliding.

A pixel-wise comparison of landslide occurrence under equivalent ground-shaking reveals two contrasting yet complementary effects (Fig. 5A). Across all hillslopes subjected to equivalent PGA in both earthquakes, the 2013 event mobilized a substantially larger area (21.21 km^2^) than the 2022 event (4.45 km^2^). This disparity is consistent with the first earthquake transporting down-slope much of the readily failure-prone material and shifting the landscape toward a more mechanically stable configuration at the regional scale. Yet, within the 2022 landslide population, new failures (3.52 km^2^; 79.18%) greatly outnumber failures overlapping 2013 landslides (0.93 km^2^; 20.82%). All 2022 failures occurred within areas that had already experienced substantial shaking during the 2013 earthquake, with PGA exceeding the cited threshold (0.12 g) for coseismic damage initiation (*32*). Remote sensing further shows that hillslopes that failed only in 2022 remained actively deforming during the interseismic period. These observations suggest that many slopes left intact in 2013 were likely preconditioned by the earlier event and therefore failed in 2022 under comparable shaking. The event-PGA interaction and the weak effect-size differences in static conditioning factors further indicate that this contrast cannot be explained by shaking intensity or preexisting slope-population differences alone (figs. S3 and S4). The first earthquake therefore both removed unstable material and created a new population of weakened hillslopes, setting up heterogeneous responses to the second event. These seemingly contradictory signatures, regional stabilization but local weakening, can be reconciled by three conceptual damage-recovery pathways under sequential seismic forcing (Fig. 5B). These pathways are data-constrained conceptual end members that synthesize the three observed failure-history categories with their associated PGA, susceptibility-transfer, and movement signatures. Each pathway expresses a distinct interplay between coseismic failure, post-seismic damage evolution, and interseismic mechanical adjustment, as follows:

1) Hillslopes that failed exclusively during the 2013 earthquake experienced intense shaking that mobilized and removed nearly all available unstable materials. The resulting collapse eliminated fragile zones and reshaped hillslopes into stronger configurations. Subsequent shaking in 2022 did not trigger renewed failure on these hillslopes, demonstrating that strong earthquakes can act as stabilizing agents.

2) Hillslopes that remained intact in 2013 nevertheless experienced subcritical mechanical damage (e.g., microcracking, changes in pore-pressure regimes, and degradation of frictional contacts) under modest shaking. Over the subsequent decade, these latent instabilities evolved through internal adjustment and hydromechanical reorganization, culminating in failure during the 2022 event under recurrent modest shaking. This pathway illustrates a time-dependent damage accumulation process, whereby hillslopes become increasingly failure-prone without initial collapse.

3) Hillslopes that failed in both earthquakes exhibit intermediate behavior. The 2013 event generated partial failures, leaving adjacent sections subcritically damaged but not mobilized. Remote-sensing observations confirm these damaged remnants continued to deform interseismically, eventually undergoing renewed failure in 2022. This two-stage failure sequence demonstrates how moderate shaking partitions a hillslope into mobilized and weakened units, the latter retaining memory of a mechanical state that governs future behavior.

Collectively, these three damage-recovery pathways describe post-seismic relaxation and synthesize the coexisting stabilizing and weakening effects of earthquakes. Intense shaking removes unstable mass; weak shaking seeds damage that evolves over time. The balance between these processes dictates how post-seismic recovery unfolds, governing the persistence and predictability of coseismic landslide hazards.

### Asymmetric predictability between sequential earthquakes

The damage-recovery pathways provide a framework for interpreting the observed directional asymmetry in cross-event susceptibility modeling. Models trained on the 2013 (predamage) landscape transfer forward successfully because they capture baseline controls (e.g., topography, lithology, and drainage) that continue to influence where mass is next available to fail. Although post-seismic weakening introduces variability, the underlying structure remains informative.

By contrast, models trained on the 2022 (post-damage) landscape capture transient damage-conditioned relationships that reflect the mechanically altered substrate, including enhanced deformation rates and modified hydrological connectivity. When applied retrospectively to 2013, these models incorrectly project the damaged state onto a landscape that had not yet experienced the same level of disturbance and therefore perform poorly.

The resulting asymmetry in model transferability is therefore interpreted as an important component of the evidence chain. Together with the PGA-failure relationships and interseismic movement observations, this asymmetry is consistent with time-dependent mechanical memory: Once damage has accumulated, susceptibility relationships evolve irreversibly through time. As we have shown, cross-event model transferability could serve as a quantitative indicator of changes in inherited hillslope condition, particularly when interpreted alongside independent constraints on post-seismic relaxation.

### A multi-timescale view of post-seismic hillslope recovery

Recent insights into the relaxation timescale draw upon seismic interferometry to suggest that mechanical damage in the shallow subsurface imparts a transient phase of enhanced landslide susceptibility. For instance, previous studies have reported a post-seismic peak in frequency of rainfall-triggered landslides that decayed to background rates after 1–4 years, a timescale that tracks the restoration of near-surface elastic properties observed in the return to pre-earthquake seismic velocities (*8*, *9*, *33*). These observations point to a short characteristic relaxation timescale of months to a maximum of a few years.

Our analysis points in a different direction. Based on cross-event model transferability, PGA-failure relationships, and sustained interseismic movement, we infer that the mechanical stress state of the hillslopes had not fully relaxed at the regional scale nearly a decade after the 2013 event. To reconcile this comparatively long relaxation, we recall the multi-timescale dynamics that are inherent to the mechanical behavior of the shallow subsurface (*7*, *34*, *35*); in short: (i) elastic crack closure, comprising the fast (<10^0^ year) recovery component; (ii) subcritical crack healing/sealing via fluid-mediated physicochemical processes; and (iii) permanent erasure of cracks via stress corrosion and pressure-solution healing/sealing processes were sustained over lengthy time spans (>10^1^ years). Recovery proxies based on seismic interferometry reflect mainly the fast component (*8*, *9*), whereas our Lushan observations fall within the slower-evolving part of the damage spectrum most relevant to transient changes in rock strength, such as persistent crack-network geometry, spatially heterogeneous weakening, and drainage disruption (*36*, *37*). In other words, different proxies track different aspects of relaxation. Considering the multi-timescale nature of damage-recovery, we propose that coseismic landslide susceptibility is a function of prolonged mechanical memory—much longer than recognized previously.

### The evolution of mountain belts

Expanding the spatial and temporal scope, prolonged mechanical memory carries broader geomorphic significance for the evolution of mountain belts. Rather than acting as isolated disturbances, coseismic landslides are components of a self-regulating system in which episodic mass wasting couples rock uplift, fluvial incision, and relief adjustment via feedbacks that set the pace and style of denudation (*38*–*40*). Sequential earthquakes do more than impose transient forcing; they repeatedly reconfigure the inherited mechanical state of hillslopes, which in turn governs whether the next shaking event meets with resilient or failure-prone hillslopes. Our three damage-recovery pathways outlined above provide an empirically-based description of hillslope memory at the event-to-decadal scale, consistent with the notion that landslide susceptibility is not solely intensity-controlled but evolves in a path-dependent manner as landscapes store, reorganize, and selectively erase damage through time (*1*, *2*).

Given their direct hand in damage-recovery processes, substrate properties (lithology, micro/macro crack density, and the chemistry of mobile fluids) determine which pathway dominates locally, thereby imparting spatial heterogeneity to both hazard and long-term denudation patterns. We show that landsliding at Lushan is concentrated preferentially on deformed metamorphic rocks, possibly reflecting that lithology and topography are often closely coupled. While topography partly controls the distribution of pore pressure and crack dilation key to frictional resistance to failure (*40*), lithology influences the chemical composition of fluids that dictate the activity of dissolution-precipitation and secondary cementation during healing/sealing (*7*). Especially influential is the density of fracturing inherited from the thermomechanical history of mountain belts during exhumation (*41*). Dense crack networks promote fluid connectivity, catalyzing healing/sealing processes and lowering the threshold for distributed crack propagation during shaking (*42*). The evolution of these crack networks at the microscale (less than a millimeter) constitutes the mechanistic bridge between short-range response of elastic recovery and the slow dynamics of rock-strength evolution. It may be that substrate properties are the primary determinant of long-term damage-recovery trajectories under recurrent earthquakes. If so, this could yield the basis of a mechanistic explanation for how substrate controls topography in seismically active mountain belts (*6*, *40*).

For geohazard science, these insights motivate a shift from static, event-specific susceptibility mapping toward dynamic, memory-aware approaches that incorporate interseismic deformation, residual damage, and recovery timescales. More broadly, by connecting earthquake sequencing, hillslope mechanics, and landscape adjustment, our findings highlight that mountain belts do not simply record seismic events; they integrate them into an evolving mechanical history that is both a response and driver of short-term hazard and long-term topographic development.

## MATERIALS AND METHODS

### Landslide inventories for the 2013 and 2022 Lushan earthquakes

We constructed polygon-based coseismic landslide inventories for the 2013 *M*_w_ 6.6 and 2022 *M*_w_ 5.8 Lushan earthquakes. Three criteria were adopted to ensure mapping reliability (*43*): (i) All landslides were delineated as polygons, (ii) polygon outlines captured the full geomorphic footprint of each landslide, and (iii) clear and complete boundaries were available.

Based on these criteria, we selected and reconciled five published inventories, three for 2013 (*20*, *22*, *23*) and two for 2022 (*21*, *24*). For each earthquake, the polygon inventories were first merged to obtain the maximum mapped landslide extent. Missing or misclassified features were corrected using an integrated workflow of landslide recognition based on change detection (*44*) and visual interpretation. SPOT 4 and aerial imagery were used for the 2013 pre- and post-earthquake comparison, PlanetScope images for the 2022 event. All mapped features were confirmed as coseismic, and any preexisting or rainfall-induced landslides were excluded (fig. S5). The resulting inventories provide a consistent, high-resolution basis for analyzing spatial pattern, scale, and morphological characteristics of landslides triggered by the two earthquakes. For cross-event comparison and susceptibility modeling, both polygon-based inventories were rasterized to a 30-m grid, matching the spatial resolution of the conditioning factors.

### Landslide susceptibility modeling using random Forest

RF was selected for susceptibility modeling due to its ability to capture nonlinear relationships and robust performance with high-dimensional inputs (fig. S5) (*45*). The RF classifier aggregates predictions from an ensemble of decision trees following (Eq. 1)

yˆ=argmaxc∑k=1Kwk·I(yˆk=c) (1)

where $\hat{y}$ denotes the final predicted class, ${\hat{y}}_{k}$ represents the class predicted by the k th tree, $w_{k}$ denotes the weight assigned to that tree, and I(·) is an indicator function that equals *1* when the k th tree predicts class c and *0* otherwise. The operator $\underset{c}{\text{argmax}}$ returns the class that maximizes the weighted sum of votes. Variable importance was quantified using the mean decrease in accuracy after random permutation of each predictor in out-of-bag samples (*46*) (Eq. 2)

$$S_{j}=\frac{1}{K}\sum_{k=1}^{K}∆{\text{MSE}}_{j}\left(k\right)$$ (2)

where $S_{j}$ represents the importance of feature j and $∆{\mathrm{MSE}}_{j}\left(k\right)$ indicates the reduction in mean squared error at the split node for feature j in the k th tree.

Before model training, multicollinearity among conditioning factors was assessed using the variance inflation factor and Pearson correlation coefficient. Highly collinear and correlated variables were removed to ensure independent predictive power (*47*, *48*). For each earthquake, we balanced the samples by pairing landslide pixels with an equal number of randomly sampled nonlandslide pixels. Negative samples were drawn from nonlandslide pixels with PGA > 0.12 g in the training event to avoid inflated accuracy from stable areas outside the shaken domain (*11*). For within-event experiments, 70% of the samples were used for training and 30% for validation, whereas for cross-event experiments, the full sample set from one earthquake was used for training and that from the other earthquake for independent testing. Each RF model consisted of 500 trees, with node splits determined by the Gini index and the number of variables considered at each split set to the square root of the total predictors (*49*). Outputs were converted to raster format to produce pixel-level susceptibility maps. Performance was assessed using AUC, *F*_1_ score, OA, and the Kappa coefficient, jointly evaluating both landslide and nonlandslide prediction. To reduce random effects from sample partitioning and model fitting, all accuracy metrics (including factor importance) were reported as the mean of 10 Monte Carlo runs.

### Interseismic hillslope movement derived from remote sensing

To quantify interseismic ground-surface movement between the 2013 and 2022 events, we combined optical image correlation for horizontal displacement with multitemporal InSAR for line-of-sight (LOS) deformation (fig. S5). The optical analysis provides the primary characterization of interseismic movement by capturing meter-scale cumulative displacement over most of the interval between the two earthquakes, matching the scale of landslide-related slope adjustment in this setting. The millimeter-scale InSAR observations, which are additionally constrained by coherence and shorter data availability windows, serve as a complementary constraint on the spatial pattern of post-seismic deformation and the contrasts among hillslopes following different damage-recovery pathways.

Horizontal displacement fields were produced using COSI-Corr (*31*), which applies normalized cross-correlation in the frequency domain for subpixel offset detection. Landsat 8 land surface reflectance images (07 December and 20 April 2022) were selected on the basis of spatial completeness, cloud-free conditions, and consistent viewing geometry. Images were orthorectified and coregistered to <0.1-pixel accuracy. Correlation window parameters were optimized through multiple trials, with a final configuration of 16 × 8 pixels yielding coherent displacement fields (signal noise ratio > 0.9). Quadratic peak fitting provided subpixel refinement. The resulting displacement fields quantify cumulative horizontal hillslope movement between the two earthquakes.

Land surface deformation was characterized using a two-tier multitemporal InSAR approach integrating persistent scatterer (PS) and distributed scatterer (DS) processing (*50*). In the first-tier network, reliable PS points were identified using amplitude dispersion and coherence thresholds and refined via a robust M-estimator with beamforming to minimize phase noise and improve relative height and velocity estimates (*51*). Statistically homogeneous pixels were then identified for DS preprocessing using the Kolmogorov-Smirnov test, and a coherence-weighted phase-linking algorithm was used for efficient phase reconstruction (*52*). In the second-tier network, deformation parameters for remaining PS and DS points were estimated through local star-shaped connections to the first-tier references, ensuring consistent velocity fields. We processed all available continuous Sentinel-1 C-band SAR data, including 170 ascending images (23 July 2016 and 29 May 2022) and 29 descending images (15 March 2017 and 10 March 2018), with a central incidence angle of 39.7°. These acquisition windows reflect the full data availability for the study area and span substantial portions of the post-seismic period (fig. S6). Topographic phase was removed using the 30-m DEM, and LOS velocities were projected onto the downslope direction to compare relative deformation patterns among hillslope groups.

## Supplementary Materials

### This PDF file includes:

Supplementary Text Figs. S1 to S6 Table S1 References
