# Supplementary material for: Hillslope memory of sequential earthquakes
Source: Sci Adv. 2026 Sep 2;12(36):eaeh3921. doi: 10.1126/sciadv.aeh3921 (PMC13537263; doi:10.1126/sciadv.aeh3921)
Supplement: Supplementary file 1 [file sciadv.aeh3921_sm.pdf]

Science Advances
AAAS

Supplementary Materials for

Hillslope memory of sequential earthquakes

Xin Wang *et al.*

Corresponding author: Xuanmei Fan, fxm_cdut@qq.com

Sci. Adv. **12**, eaeh3921 (2026)
DOI: 10.1126/sciadv.aeh3921

This PDF file includes:

Supplementary Text
Figs. S1 to S6
Table S1
References

Supplementary Text

To characterize the controls on coseismic landsliding, we selected fifteen factors commonly used in earthquake-triggered landslide studies. Topographic factors, including altitude, slope, aspect, curvature, and TPI, were derived from the NASADEM (*53*) using TopoToolbox (*54*), while landform categories were obtained following (*55*). Geological attributes, including lithology and soil type, were extracted from the GLiM (*56*) and FAO Digital Soil Map of the World (*57*). Fault traces were obtained from CAFD (*58*). Land cover was taken from the temporally closest pre-event GLC_FCS30D product (*59*), while NDVI was computed from Landsat 8 pre-earthquake imagery. Since small tributaries are often missing in global datasets, hydrological indicators including distance to rivers and TWI were derived from DEM-based river extraction and catchment modelling using TopoToolbox. Seismic shaking parameters (PGA, PGV) were obtained from USGS ShakeMap products (latest versions) (*60*). The archived products of conditioning factors for the 2013 and 2022 earthquakes were rasterized and projected to the 30 m grid, and grid-cell values were then extracted for each landslide and non-landslide pixel. Iterated multicollinearity analysis and Pearson's correlation validation were implemented to remove variables with strong redundancy, leading to exclusion of PGV and TPI due to high correlations with PGA and curvature, respectively.

Both earthquakes show strong proximity effects: 97.4%±1.5% of landslides occurred within 7 km of major faults, and 96.1%±2.3% within 3 km of rivers. This consistent decay with distance reflects long-term tectonic weakening near active structures and enhanced incision-driven instability along river valleys (*5, 61, 62*). Slope angle and elevation exhibit peaked frequency distributions, with failures concentrated within specific ranges rather than increasing monotonically. This pattern suggests that only slopes exceeding a certain critical steepness or weathering state become responsive to seismic forcing (*4, 63, 64*). PGA shows a comparable threshold behaviour: 78.3% (2013) and 88.6% (2022) of landslides fall within 0.16-0.34 g and 0.16-0.24 g, respectively, confirming that ground acceleration exerted strong, consistent controls on both events.

TPI, TWI, and curvature display similar trends, with landslides clustering near valley bottoms, steep slopes, and convergent terrain. These features enhance hydrological concentration and gravitational stresses, thereby conditioning slopes for failure (*65, 66*). NDVI values indicate that 98.9%±0.2% of landslides occurred in areas with moderate-to-high vegetation cover (0.5-1.0). While vegetation typically contributes to shallow reinforcement, its presence in humid, weathered landscapes may also reflect long-term slope weakening, explaining its positive association with landslide occurrence (*67, 68*). Both earthquakes preferentially affected metamorphic lithologies and lithosols, whose mechanical properties, such as fracturing, low cohesion, and limited soil development, make them inherently susceptible (*69, 70*). Shrubland and forest were the dominant land cover types at failed locations, consistent with their steep terrain and locally unstable soil horizons.

Landform classification, integrating temperature and topographic context (*55*), shows that landslides were more concentrated on lower-slope landforms and on cooler aspects. These areas tend to exhibit enhanced moisture retention, intensified freeze-thaw weathering, and greater accumulation of weathered material (*71, 72*), favoring slip surface development. Aspect displayed the most pronounced contrast between the two earthquakes. In the NE direction (22.5°-

67.5°), the 2013 earthquake produced the lowest landslide density whereas the 2022 event exhibited the highest. Conversely, W-SW-S-SE-facing slopes hosted the most failures in 2013 but the fewest in 2022. This reversal aligns with the distinct rupture geometries and shaking directivities of the two events, which redistributed seismic energy across slopes in different orientations (*65*). Despite these directional differences, the overall controlling factors remained consistent across both earthquakes under the same environmental setting.

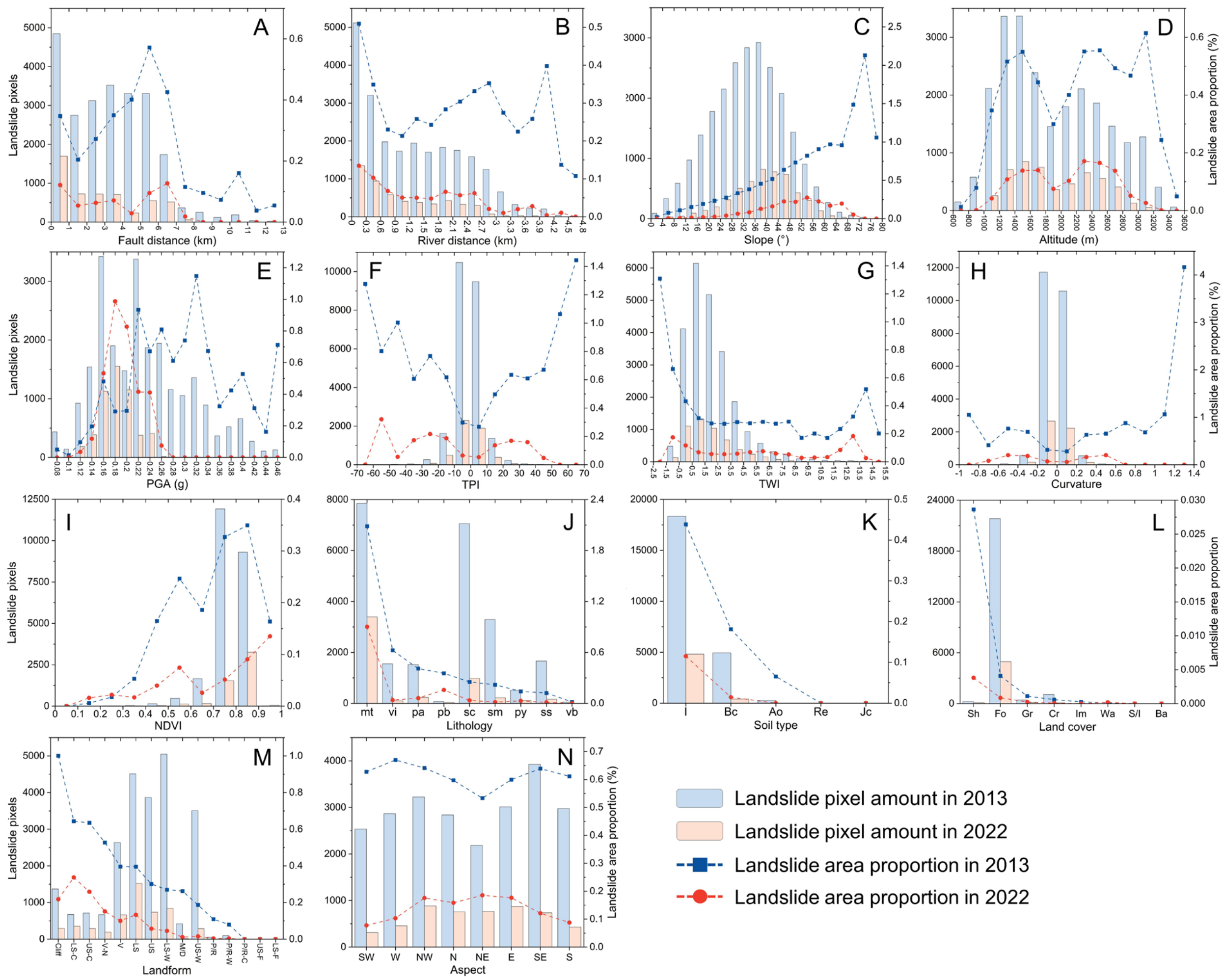

Fig. S1.

Spatial patterns of coseismic landslides across conditioning factors. Spatial distributions of landslides as a function of major conditioning factors: (**A**) distance to fault, (**B**) distance to river, (**C**) slope, (**D**) altitude, (**E**) PGA, (**F**) TPI, (**G**) TWI, (**H**) curvature, (**I**) NDVI, (**J**) lithology, (**K**) soil type, (**L**) land cover, (**M**) landform, (**N**) aspect. Lithology abbreviations: mt = metamorphics, vi = intermediate volcanic rocks, pa = acid plutonic rocks, pb = basic plutonic rocks, sc = carbonate sedimentary rocks, sm = mixed sedimentary rocks, py = pyroclastics, ss = siliciclastic sedimentary rocks, vb = basic volcanic rocks. Soil type abbreviations: l = lithosols, Bc = chromic cambisols, Ao = orthic acrisols, Re = eutric gleysols, Jc = calcaric fluvisols. Land cover abbreviations: Sh = shrub, Fo = forest, Gr = grassland, Cr = cropland, Im = impervious surface, Wa = water, S/I = snow/ice, Ba = Barren. Landform abbreviations: LS-C = lower slope (cool), US-C = upper slope (cool), V-N = valley (narrow), LS = lower slope, US = upper slope, LS-W = lower slope (warm), M/D = mountain/divide, US-W = upper slope (warm), P/R = peak/ridge, P/R-W = peak/ridge (warm), P/R-C = peak/ridge (cool), LS-F = lower slope (flat), US-F = upper slope (flat).

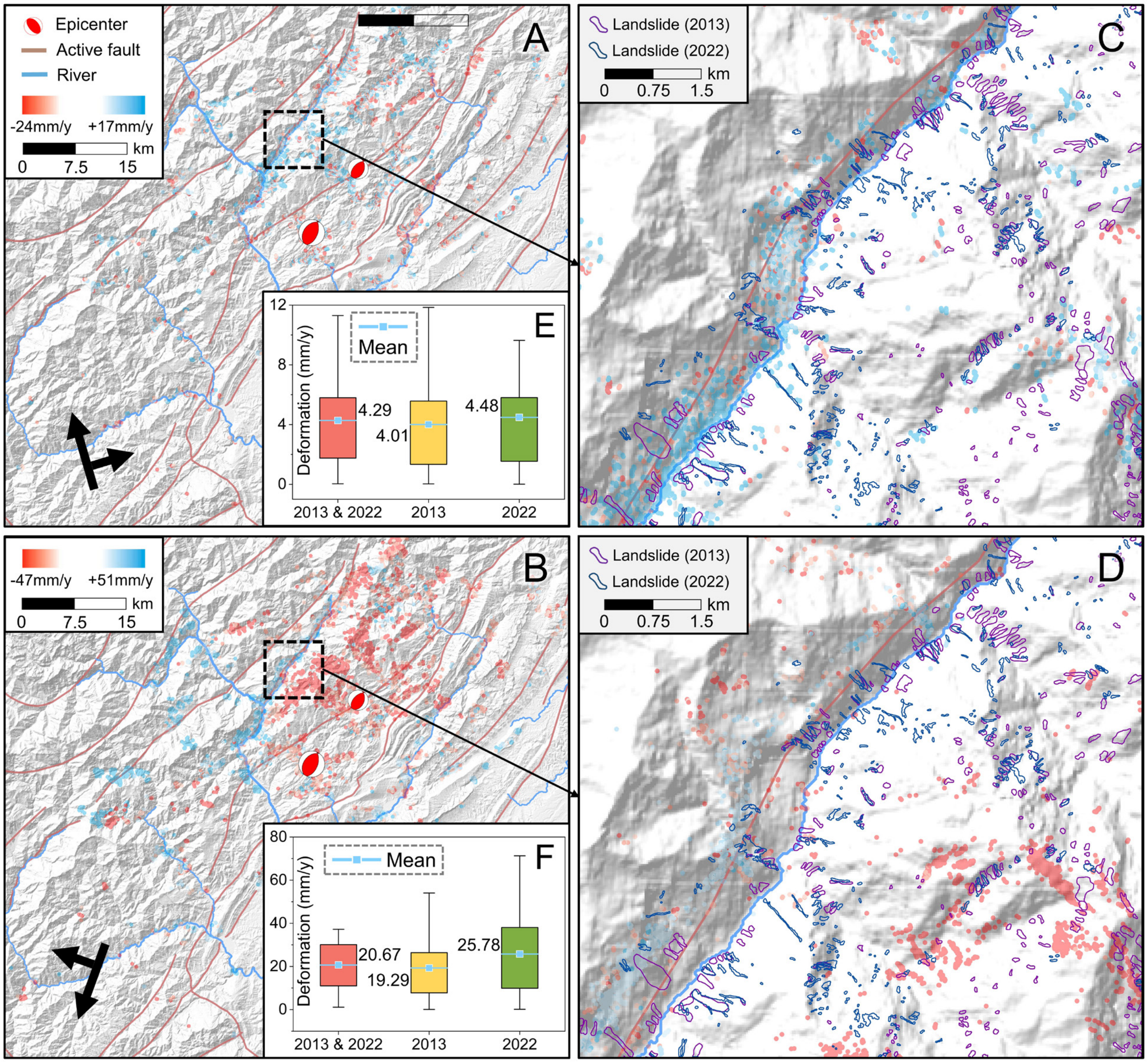

Fig. S2.

Interseismic slope deformation between the two earthquakes. Line-of-sight deformation velocity fields retrieved from Sentinel-1 MT-InSAR processing: (**A**) ascending (2016-07-23 to 2022-05-29) and (**B**) descending (2017-03-15 to 2018-03-10) solutions. Enlarged view of (**C**) ascending and (**D**) descending hillslope deformation where coseismic landslides were most densely concentrated. Deformation statistics for slopes that experienced different landslide categories from (**E**) ascending and (**F**) descending solutions.

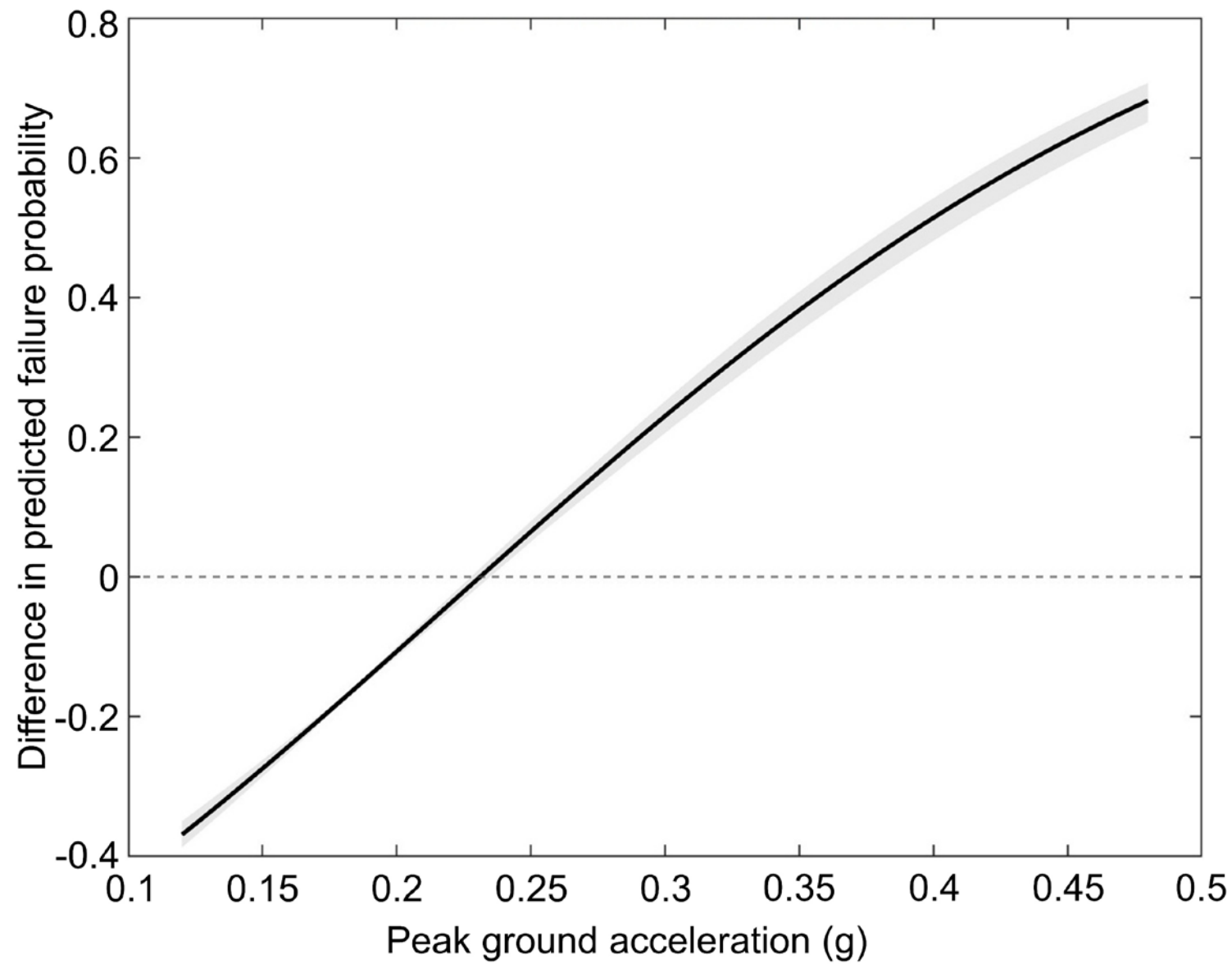

Fig. S3.

Event-PGA interaction analysis based on the difference in landslide failure probability between the 2022 and 2013 earthquakes. The difference curve is derived from a logistic binomial model of the landslide probability as a function of event and PGA, using a two-way interactions model. The event-PGA interaction coefficient was consistently positive ($\beta = 14.03 \pm 0.52$) and remained significant ($p < 0.05$), indicating the expected landslide response to ground shaking was systematically different between the two events. Positive values indicate that, at the same PGA, the predicted failure probability is higher in 2022 than in 2013, whereas negative values indicate the opposite. The shaded interval represents 95% confidence bounds. This event-PGA interaction analysis provides important evidence suggesting that the later hillslope response cannot be explained by contemporaneous shaking intensity alone, and instead supports a time-dependent inherited influence of the earlier earthquake on the later coseismic failure.

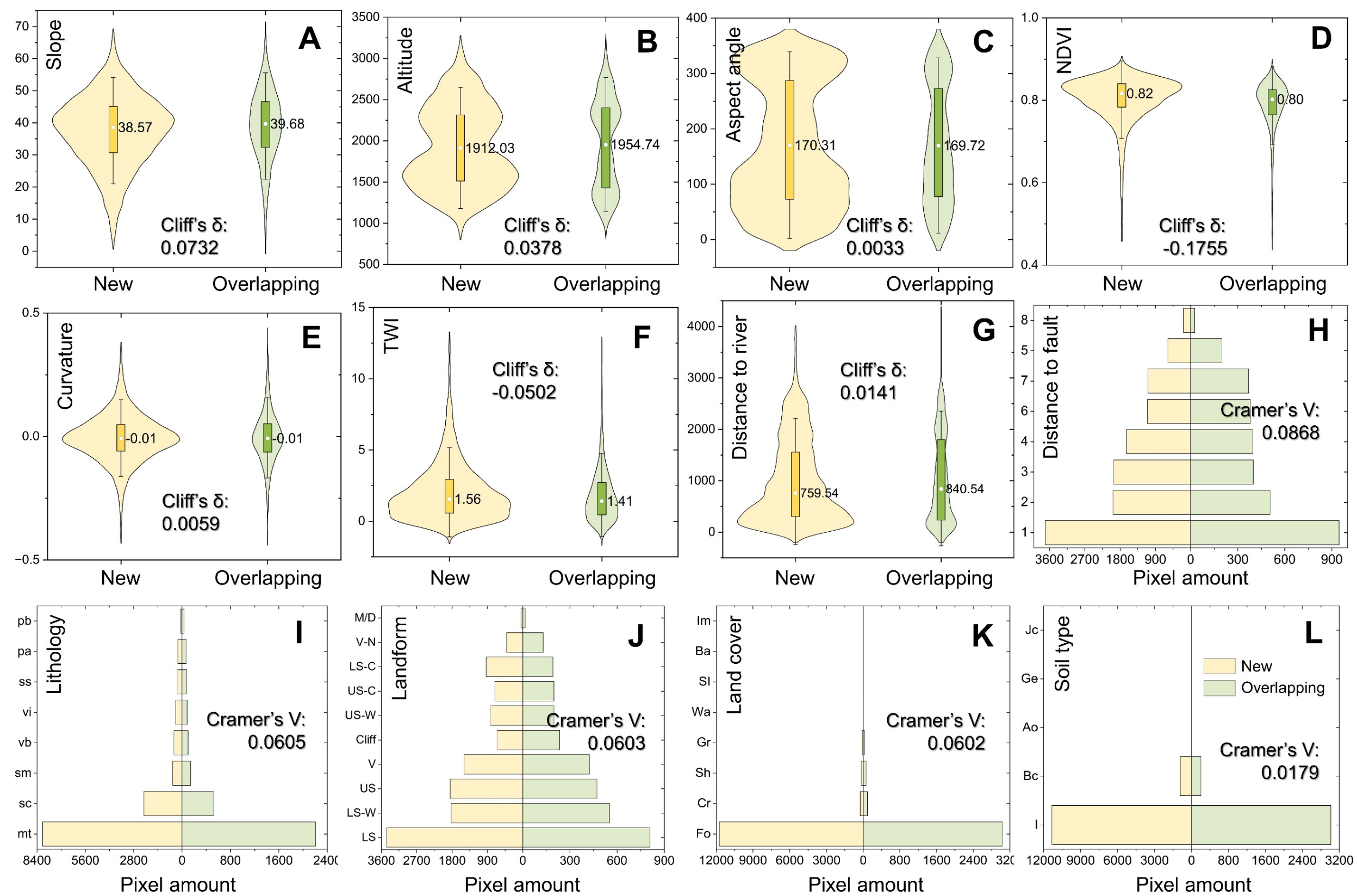

Fig. S4.

Comparison of factor distribution between 2022 newly triggered failures and 2022 failures overlapping 2013 landslides. Distributions of continuous static conditioning factors, including slope (**A**), altitude (**B**), aspect (**C**), NDVI (**D**), curvature (**E**), TWI (**F**), and distance to river (**G**). White dots denote medians and boxes denote interquartile ranges. Cliff's δ is reported as the effect size. Frequency distributions of binned and categorical static conditioning factors, including distance to fault (**H**), lithology (**I**), landform (**J**), land cover (**K**), and soil type (**L**). Cramer's V is reported as the effect size. The two groups comprise 12,253 pixels of newly triggered failures and 3,219 pixels of failures overlapping 2013 landslides. Overall, effect sizes are weak across the static conditioning factors, indicating that the two categories are not strongly separated by pre-existing geomorphic, geological, or environmental settings. The relatively larger NDVI contrast likely reflects vegetation disturbance caused by 2013 landsliding and incomplete recovery before the 2022 earthquake. Lithology abbreviations: mt = metamorphics, vi = intermediate volcanic rocks, pa = acid plutonic rocks, pb = basic plutonic rocks, sc = carbonate sedimentary rocks, sm = mixed sedimentary rocks, py = pyroclastics, ss = siliciclastic sedimentary rocks, vb = basic volcanic rocks. Soil type abbreviations: l = lithosols, Bc = chromic cambisols, Ao = orthic acrisols, Re = eutric gleysols, Jc = calcaric fluvisols. Land cover abbreviations: Sh = shrub, Fo = forest, Gr = grassland, Cr = cropland, Im = impervious surface, Wa = water, S/I = snow/ice, Ba = barren. Landform abbreviations: LS-C = lower slope (cool), US-C = upper slope (cool), V-N = valley (narrow), LS = lower slope, US = upper slope, LS-W = lower slope (warm), M/D = mountain/divide, US-W = upper slope (warm), P/R = peak/ridge, P/R-W = peak/ridge (warm), P/R-C = peak/ridge (cool), LS-F = lower slope (flat), US-F = upper slope (flat).

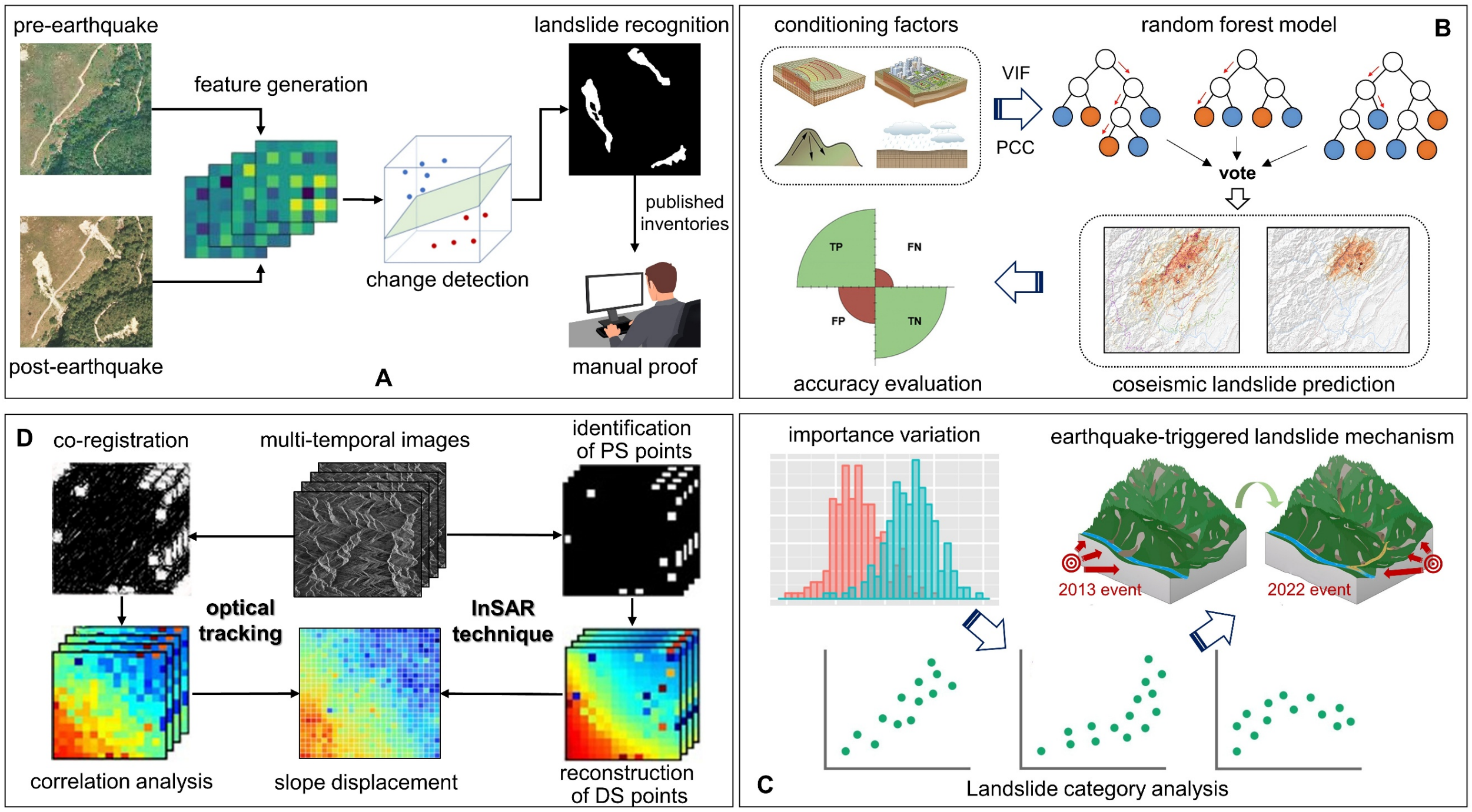

Fig. S5.

Overview of the analytical workflow. Schematic illustrating the major components of the study: (**A**) construction and refinement of high-resolution coseismic landslide inventories; (**B**) coseismic landslide susceptibility modeling using Random Forest under multiple training-prediction scenarios; (**C**) identification of the mechanical pathways governing slope evolution under repeated seismic forcing; (**D**) derivation of interseismic slope response from optical correlation and MT-InSAR observations.

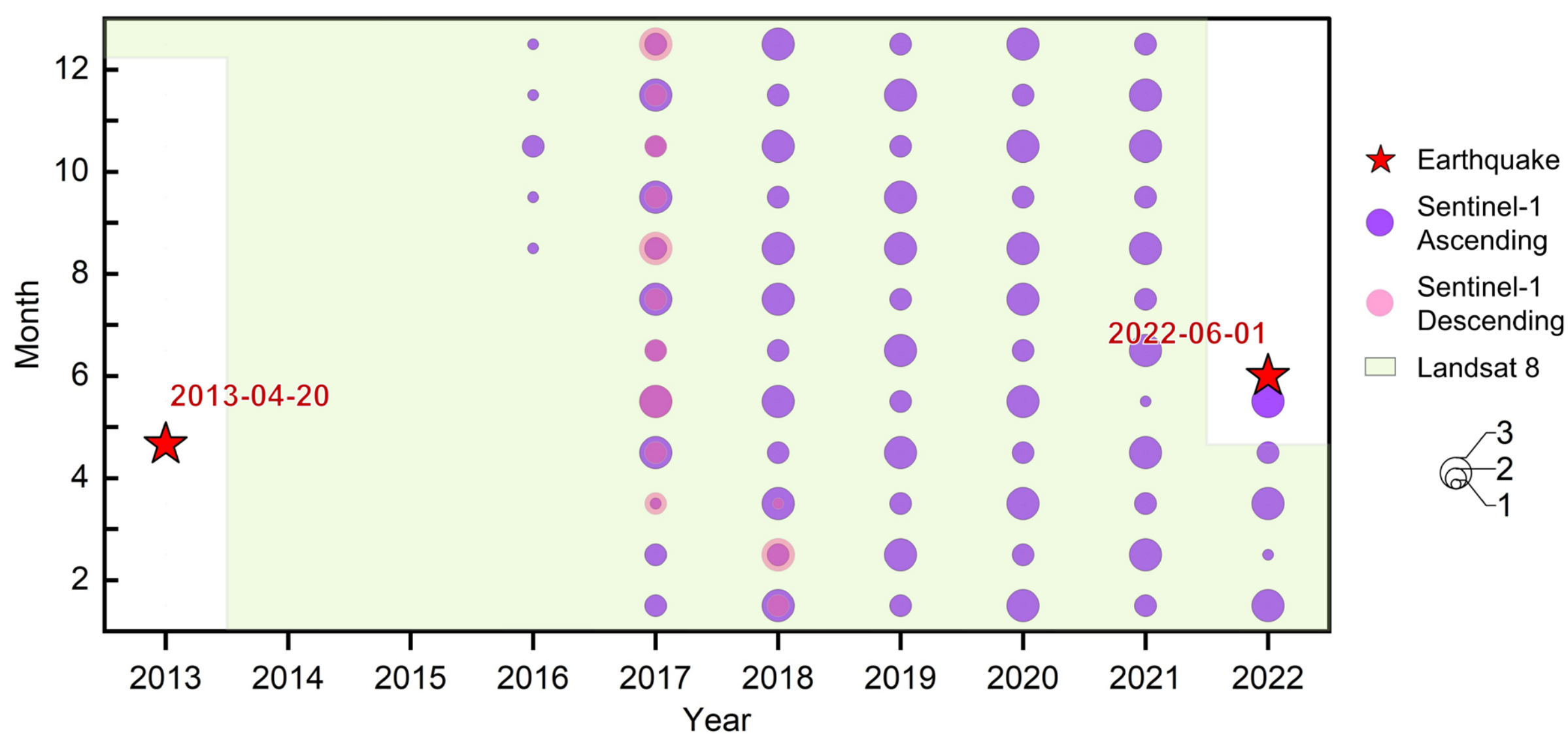

Fig. S6.

Temporal coverage of optical and SAR observations used to quantify interseismic hillslope movement between the 2013 and 2022 Lushan earthquakes.

Table S1. Summary statistics of the landslide inventories and cross-event failure categories of the recurrent Lushan earthquakes.

Category	Name	Pixels (30 m)	Area (km^2)
Landslide inventory	2013 coseismic landslides	24,569	22.11
	2022 coseismic landslides	5,623	5.06
Landslide types	Landslides failed only in 2013	23,394	21.05
	Landslides failed only in 2022	4,448	4.00
	Landslide failures overlapped in both earthquakes	1,175	1.06

REFERENCES

1. K. Beven, What we see now: Event-persistence and the predictability of hydro-eco-geomorphological systems. *Ecol. Model.* **298**, 4–15 (2015).

2. J. D. Phillips, Deterministic chaos and historical geomorphology: A review and look forward. *Geomorphology* **76**, 109–121 (2006).

3. J. S. Kargel, G. J. Leonard, D. H. Shugar, U. K. Haritashya, A. Bevington, E. J. Fielding, K. Fujita, M. Geertsema, E. S. Miles, J. Steiner, E. Anderson, S. Bajracharya, G. W. Bawden, D. F. Breashears, A. Byers, B. Collins, M. R. Dhital, A. Donnellan, T. L. Evans, M. L. Geai, M. T. Glasscoe, D. Green, D. R. Gurung, R. Heijenk, A. Hilborn, K. Hudnut, C. Huyck, W. W. Immerzeel, J. Liming, R. Jibson, A. Kääb, N. R. Khanal, D. Kirschbaum, P. D. A. Kraaijenbrink, D. Lamsal, L. Shiyin, L. Mingyang, D. Mc Kinney, N. K. Nahirnick, N. Zhuotong, S. Ojha, J. Olsenholler, T. H. Painter, M. Pleasants, K. C. Pratima, Q. I. Yuan, B. H. Raup, D. Regmi, D. R. Rounce, A. Sakai, S. Donghui, J. M. Shea, A. B. Shrestha, A. Shukla, D. Stumm, M. van der Kooij, K. Voss, W. Xin, B. Weihs, D. Wolfe, W. Lizong, Y. Xiaojun, M. R. Yoder, N. Young, Geomorphic and geologic controls of geohazards induced by Nepal's 2015 Gorkha earthquake. *Science* **351**, aac8353 (2016).

4. X. Fan, G. Scaringi, O. Korup, A. J. West, C. J. van Westen, H. Tanyas, N. Hovius, T. C. Hales, R. W. Jibson, K. E. Allstadt, S. G. Evans, C. Xu, G. Li, X. Pei, Q. Xu, R. Huang, Earthquake-induced chains of geologic hazards: Patterns, mechanisms, and impacts. *Rev. Geophys.* **57**, 421–503 (2019).

5. B. D. Malamud, D. L. Turcotte, F. Guzzetti, P. Reichenbach, Landslides, earthquakes, and erosion. *Earth Planet. Sci. Lett.* **229**, 45–59 (2004).

6. J. Wang, J. D. Howarth, E. L. McClymont, A. L. Densmore, S. J. Fitzsimons, T. Croissant, D. R. Gröcke, M. D. West, E. L. Harvey, N. V. Frith, M. H. Garnett, R. G. Hilton, Long-term patterns of hillslope erosion by earthquake-induced landslides shape mountain landscapes. *Sci. Adv.* **6**, eaaz6446 (2020).

7. N. Brantut, Time-dependent recovery of microcrack damage and seismic wave speeds in deformed limestone. *J. Geophys. Res. Solid. Earth* **120**, 8088–8109 (2015).

8. L. Illien, J. M. Turowski, C. Sens-Schönfelder, C. Berenfeld, N. Hovius, Predictable recovery rates in near-surface materials after earthquake damage. *Nat. Commun.* **16**, 1790 (2025).

9. O. Marc, C. Sens-Schönfelder, L. Illien, P. Meunier, M. Hobiger, K. Sawazaki, C. Rault, N. Hovius, Toward using seismic interferometry to quantify landscape mechanical variations after earthquakes. *Bull. Seismol. Soc. Am.* **111**, 1631–1649 (2021).

10. J. D. Howarth, N. C. Barth, S. J. Fitzsimons, K. Richards-Dinger, K. J. Clark, G. P. Biasi, U. A. Cochran, R. M. Langridge, K. R. Berryman, R. Sutherland, Spatiotemporal clustering of great earthquakes on a transform fault controlled by geometry. *Nat. Geosci.* **14**, 314–320 (2021).

11. X. Fan, X. Wang, C. Fang, J. D. Jansen, L. Dai, H. Tanyas, N. Zang, R. Tang, Q. Xu, R. Huang, Deep learning can predict global earthquake-triggered landslides. *Natl. Sci. Rev.* **12**, nwaf179 (2025).

12. Q. He, M. Wang, K. Liu, Rapidly assessing earthquake-induced landslide susceptibility on a global scale using random forest. *Geomorphology* **391**, 107889 (2021).

13. R. Parker, G. Hancox, D. Petley, C. Massey, A. Densmore, N. Rosser, Spatial distributions of earthquake-induced landslides and hillslope preconditioning in the northwest South Island, New Zealand. *Earth Surf. Dyn.* **3**, 501–525 (2015).

14. M. P. Dahlquist, A. J. West, Initiation and runout of post-seismic debris flows: Insights from the 2015 Gorkha earthquake. *Geophys. Res. Lett.* **46**, 9658–9668 (2019).

15. J. N. Jones, S. J. Boulton, G. L. Bennett, M. Stokes, M. R. Whitworth, Temporal variations in landslide distributions following extreme events: Implications for landslide susceptibility modeling. *J. Geophys. Res. Earth Surf.* **126**, e2021JF006067 (2021).

16. M. J. Brain, N. J. Rosser, N. Tunstall, The control of earthquake sequences on hillslope stability. *Geophys. Res. Lett.* **44**, 865–872 (2017).

17. P. Guo, Z. Han, C. Zhou, H. Gai, P. Niu, X. Zhang, Multi-segment ruptures of the 2023 Mw 6.0 Jishishan earthquake, Tibetan Plateau: Implications for seismogenic mechanisms of moderate earthquakes. *J. Geophys. Res. Solid. Earth* **130**, e2024JB029368 (2025).

18. Y. Li, D. Jia, M. Wang, J. H. Shaw, J. He, A. Lin, L. Xiong, G. Rao, Structural geometry of the source region for the 2013 Mw 6.6 Lushan earthquake: Implication for earthquake hazard assessment along the Longmen Shan. *Earth Planet. Sci. Lett.* **390**, 275–286 (2014).

19. K. Dai, R. Guo, X. Zou, X. Tang, X. Xu, D. Liu, Y. Zheng, J. Xu, The source characteristics of the 2022 M_s 6.1 Lushan Earthquake: An event that occurred on the conjugated fault of the 2013 M_s 7.0 Lushan Earthquake. *Seismol. Res. Lett.* **94**, 2181–2191 (2023).

20. M. Chang, P. Cui, L. Xu, Y. Zhou, The spatial distribution characteristics of coseismic landslides triggered by the Ms7.0 Lushan earthquake and Ms7.0 Jiuzhaigou earthquake in southwest China. *Environ. Sci. Pollut. Res.* **28**, 20549–20569 (2021).

21. X. Shao, C. Xu, S. Ma, Preliminary analysis of coseismic landslides induced by the 1 June 2022 Ms 6.1 Lushan Earthquake, China. *Sustainability* **14**, 16554 (2022).

22. C. Tang, G. Ma, M. Chang, W. Li, D. Zhang, T. Jia, Z. Zhou, Landslides triggered by the 20 April 2013 Lushan earthquake, Sichuan province, China. *Eng. Geol.* **187**, 45–55 (2015).

23. C. Xu, X. Xu, J. B. H. Shyu, Database and spatial distribution of landslides triggered by the Lushan, China Mw 6.6 earthquake of 20 April 2013. *Geomorphology* **248**, 77–92 (2015).

24. B. Zhao, W. Li, L. Su, Y. Wang, H. Wu, Insights into the landslides triggered by the 2022 Lushan Ms 6.1 earthquake: Spatial distribution and controls. *Remote Sens.* **14**, 4365 (2022).

25. R. Wen, Y. Ren, Strong-motion observations of the Lushan earthquake on 20 April 2013. *Seismol. Res. Lett.* **85**, 1043–1055 (2014).

26. I. J. Larsen, D. R. Montgomery, Landslide erosion coupled to tectonics and river incision. *Nat. Geosci.* **5**, 468–473 (2012).

27. G. Li, A. J. West, A. L. Densmore, Z. Jin, F. Zhang, J. Wang, M. Clark, R. G. Hilton, Earthquakes drive focused denudation along a tectonically active mountain front. *Earth Planet. Sci. Lett.* **472**, 253–265 (2017).

28. D. W. Burbank, J. Leland, E. Fielding, R. S. Anderson, N. Brozovic, M. R. Reid, C. Duncan, Bedrock incision, rock uplift and threshold hillslopes in the northwestern Himalayas. *Nature* **379**, 505–510 (1996).

29. M. Bordoni, A. Cislaghi, A. Vercesi, G. Bischetti, C. Meisina, Effects of plant roots on soil shear strength and shallow landslide proneness in an area of northern Italian Apennines. *Bull. Eng. Geol. Environ.* **79**, 3361–3381 (2020).

30. M. J. Brain, S. Moya, M. E. Kincey, N. Tunstall, D. N. Petley, S. A. Sepúlveda, Controls on post-seismic landslide behavior in brittle rocks. *J. Geophys. Res. Earth Surf.* **126**, e2021JF006242 (2021).

31. S. Leprince, S. Barbot, F. Ayoub, J.-P. Avouac, Automatic and precise orthorectification, coregistration, and subpixel correlation of satellite images, application to ground deformation measurements. *IEEE Trans. Geosci. Remote Sensing* **45**, 1529–1558 (2007).

32. R. W. Jibson, E. L. Harp, Ground motions at the outermost limits of seismically triggered landslides. *Bull. Seismol. Soc. Am.* **106**, 708–719 (2016).

33. O. Marc, N. Hovius, P. Meunier, T. Uchida, S. Hayashi, Transient changes of landslide rates after earthquakes. *Geology* **43**, 883–886 (2015).

34. C. K. Lieou, E. G. Daub, R. E. Ecke, P. A. Johnson, Slow dynamics and strength recovery in unconsolidated granular Earth materials: A mechanistic theory. *J. Geophys. Res. Solid. Earth* **122**, 7573–7583 (2017).

35. G. Pozzi, M. M. Scuderi, E. Tinti, M. Nazzari, C. Collettini, The role of fault rock fabric in the dynamics of laboratory faults. *J. Geophys. Res. Solid. Earth* **127**, e2021JB023779 (2022).

36. K. He, Y. Li, G. Ma, X. Hu, B. Liu, Z. Ma, Z. Xu, Failure mode analysis of post-seismic rockfall in shattered mountains exemplified by detailed investigation and numerical modelling. *Landslides* **18**, 425–446 (2021).

37. C. Y. Wang, A new mechanism for earthquake-enhanced permeability. *Water Resour. Res.* **58**, e2021WR031503 (2022).

38. D. L. Egholm, M. F. Knudsen, M. Sandiford, Lifespan of mountain ranges scaled by feedbacks between landsliding and erosion by rivers. *Nature* **498**, 475–478 (2013).

39. O. Korup, A. L. Densmore, F. Schlunegger, The role of landslides in mountain range evolution. *Geomorphology* **120**, 77–90 (2010).

40. G. K. Li, S. Moon, Topographic stress control on bedrock landslide size. *Nat. Geosci.* **14**, 307–313 (2021).

41. P. Molnar, R. S. Anderson, S. P. Anderson, Tectonics, fracturing of rock, and erosion. *J. Geophys. Res. Earth Surf.* **112**, e2005JF000433 (2007).

42. Q. Lei, N. G. Doonechaly, C.-F. Tsang, Modelling fluid injection-induced fracture activation, damage growth, seismicity occurrence and connectivity change in naturally fractured rocks. *Int. J. Rock Mech. Min. Sci.* **138**, 104598 (2021).

43. M. Nowicki Jessee, M. W. Hamburger, K. Allstadt, D. J. Wald, S. M. Robeson, H. Tanyas, M. Hearne, E. M. Thompson, A global empirical model for near-real-time assessment of seismically induced landslides. *J. Geophys. Res. Earth Surf.* **123**, 1835–1859 (2018).

44. X. Wang, X. Fan, Q. Xu, P. Du, Change detection-based co-seismic landslide mapping through extended morphological profiles and ensemble strategy. *ISPRS J. Photogramm. Remote Sens.* **187**, 225–239 (2022).

45. M. Belgiu, L. Drăguţ, Random forest in remote sensing: A review of applications and future directions. *ISPRS J. Photogramm. Remote Sens.* **114**, 24–31 (2016).

46. V. F. Rodriguez-Galiano, B. Ghimire, J. Rogan, M. Chica-Olmo, J. P. Rigol-Sanchez, An assessment of the effectiveness of a random forest classifier for land-cover classification. *ISPRS J. Photogramm. Remote Sens.* **67**, 93–104 (2012).

47. P. Ahlgren, B. Jarneving, R. Rousseau, Requirements for a cocitation similarity measure, with special reference to Pearson's correlation coefficient. *J. Am. Soc. Inf. Sci. Technol.* **54**, 550–560 (2003).

48. C. F. Dormann, J. Elith, S. Bacher, C. Buchmann, G. Carl, G. Carré, J. R. G. Marquéz, B. Gruber, B. Lafourcade, P. J. Leitão, T. Münkemüller, C. McClean, P. E. Osborne, B. Reineking, B. Schröder, A. K. Skidmore, D. Zurell, S. Lautenbach, Collinearity: A review of methods to deal with it and a simulation study evaluating their performance. *Ecography* **36**, 27–46 (2013).

49. M. Pal, Random forest classifier for remote sensing classification. *Int. J. Remote Sens.* **26**, 217–222 (2005).

50. B. Zhang, R. Wang, Y. Deng, P. Ma, H. Lin, J. Wang, Mapping the Yellow River Delta land subsidence with multitemporal SAR interferometry by exploiting both persistent and distributed scatterers. *ISPRS J. Photogramm. Remote Sens.* **148**, 157–173 (2019).

51. P. Ma, H. Lin, Robust detection of single and double persistent scatterers in urban built environments. *IEEE Trans. Geosci. Remote Sensing* **54**, 2124–2139 (2015).

52. N. Cao, H. Lee, H. C. Jung, Mathematical framework for phase-triangulation algorithms in distributed-scatterer interferometry. *IEEE Geosci. Remote Sens. Lett.* **12**, 1838–1842 (2015).

53. R. Crippen, S. Buckley, P. Agram, E. Belz, E. Gurrola, S. Hensley, M. Kobrick, M. Lavalle, J. Martin, M. Neumann, Q. Nguyen, P. Rosen, J. Shimada, M. Simard, W. Tung, NASADEM global elevation model: Methods and progress. *Int. Arch. Photogramm. Remote Sens. Spatial Inf. Sci.* **XLI-B4**, 125–128 (2016).

54. W. Schwanghart, D. Scherler, TopoToolbox 2–MATLAB-based software for topographic analysis and modeling in Earth surface sciences. *Earth Surf. Dyn.* **2**, 1–7 (2014).

55. D. M. Theobald, D. Harrison-Atlas, W. B. Monahan, C. M. Albano, Ecologically-relevant maps of landforms and physiographic diversity for climate adaptation planning. *PLOS ONE* **10**, e0143619 (2015).

56. J. Hartmann, N. Moosdorf, The new global lithological map database GLiM: A representation of rock properties at the Earth surface. *Geochem. Geophys. Geosyst.* **13**, e2012GC004370 (2012).

57. P. A. Sanchez, S. Ahamed, F. Carré, A. E. Hartemink, J. Hempel, J. Huising, P. Lagacherie, A. B. McBratney, N. J. McKenzie, M. D. L. Mendonça-Santos, B. Minasny, L. Montanarella, P. Okoth, C. A. Palm, J. D. Sachs, K. D. Shepherd, T. Vågen, B. Vanlauwe, M. G. Walsh, L. A. Winowiecki, G. Zhang, Digital soil map of the world. *Science* **325**, 680–681 (2009).

58. X. Wu, X. Xu, G. Yu, J. Ren, X. Yang, G. Chen, C. Xu, K. Du, X. Huang, H. Yang, K. Li, H. Hao, The China Active Faults Database (CAFD) and its web system. *Earth Syst. Sci. Data* **16**, 3391–3417 (2024).

59. X. Zhang, T. Zhao, H. Xu, W. Liu, J. Wang, X. Chen, L. Liu, GLC_FCS30D: The first global 30 m land-cover dynamics monitoring product with a fine classification system for the period from 1985 to 2022 generated using dense-time-series Landsat imagery and the continuous change-detection method. *Earth Syst. Sci. Data* **16**, 1353–1381 (2024).

60. D. J. Wald, C. B. Worden, E. M. Thompson, M. Hearne, ShakeMap operations, policies, and procedures. *Earthq. Spectra* **38**, 756–777 (2022).

61. M. J. Crozier, Deciphering the effect of climate change on landslide activity: A review. *Geomorphology* **124**, 260–267 (2010).

62. G. Scaringi, M. Loche, A thermo-hydro-mechanical approach to soil slope stability under climate change. *Geomorphology* **401**, 108108 (2022).

63. F. Dai, C. F. Lee, Y. Y. Ngai, Landslide risk assessment and management: An overview. *Eng. Geol.* **64**, 65–87 (2002).

64. A. I. Patton, S. L. Rathburn, D. M. Capps, D. McGrath, R. A. Brown, Ongoing landslide deformation in thawing permafrost. *Geophys. Res. Lett.* **48**, e2021GL092959 (2021).

65. P. Meunier, N. Hovius, J. A. Haines, Topographic site effects and the location of earthquake induced landslides. *Earth Planet. Sci. Lett.* **275**, 221–232 (2008).

66. D. R. Montgomery, W. E. Dietrich, A physically based model for the topographic control on shallow landsliding. *Water Resour. Res.* **30**, 1153–1171 (1994).

67. S. Bordoloi, C. W. W. Ng, The effects of vegetation traits and their stability functions in bio-engineered slopes: A perspective review. *Eng. Geol.* **275**, 105742 (2020).

68. M. T. Löbmann, C. Geitner, C. Wellstein, S. Zerbe, The influence of herbaceous vegetation on slope stability–a review. *Earth Sci. Rev.* **209**, 103328 (2020).

69. L. Fan, P. Lehmann, D. Or, Effects of soil spatial variability at the hillslope and catchment scales on characteristics of rainfall-induced landslides. *Water Resour. Res.* **52**, 1781–1799 (2016).

70. F. Guzzetti, M. Cardinali, P. Reichenbach, The influence of structural setting and lithology on landslide type and pattern. *Environ. Eng. Geosci.* **2**, 531–555 (1996).

71. M. Deprez, T. De Kock, G. De Schutter, V. Cnudde, A review on freeze-thaw action and weathering of rocks. *Earth Sci. Rev.* **203**, 103143 (2020).

72. R. M. Iverson, Landslide triggering by rain infiltration. *Water Resour. Res.* **36**, 1897–1910 (2000).
